# Effect of oxygen supplementation on cognitive performance among HEMS providers after acute exposure to altitude: the HEMS II randomized clinical trial

**DOI:** 10.1186/s13049-024-01238-6

**Published:** 2024-07-29

**Authors:** Marika Falla, Michiel J. van Veelen, Markus Falk, Elisabeth M. Weiss, Giulia Roveri, Michela Masè, Bernhard Weber, Anna Randi, Hermann Brugger, Katharina Hüfner, Giacomo Strapazzon

**Affiliations:** 1 Institute of Mountain Emergency Medicine, Eurac Research, Bolzano, Italy; 2 Department of Neurology/Stroke Unit, Hospital of Bolzano (SABES-ASDAA), Bolzano, Italy; 3 Lehrkrankenhaus der Paracelsus Medizinischen Privatuniversität, Salzburg, Austria; 4 Center for Mind/Brain Sciences, University of Trento, Rovereto, Italy; 5 Department of Sport Science, Medical Section, University of Innsbruck, Innsbruck, Austria; 6 Institute of Psychology, University of Innsbruck, Innsbruck, Austria; 7 Department of Anaesthesiology and Intensive Care Medicine, Hospital of Merano (SABES-ASDAA), Merano, Italy; 8 Department of Cellular, Computational, and Integrative Biology, CIBIO, Trento, Italy; 9 Department of Psychology, University of Graz, Graz, Austria; 10 Division of Psychiatry II, Department of Psychiatry, Psychotherapy, Psychosomatics and Medical Psychology, Medical University of Innsbruck, Innsbruck, Austria

**Keywords:** Oxygen supplementation, Cognition, Altitude, Hypobaric hypoxia, Brain, Attention, Reaction time, Working memory, Processing speed, Motor skills, Stress, Workload, Helicopter emergency medical services

## Abstract

**Importance:**

Emergency medical services (EMS) providers transiently ascend to high altitude for primary missions and secondary transports in mountainous areas in helicopters that are unpressurised and do not have facilities for oxygen supplementation. The decrease in cerebral oxygen saturation can lead to impairment in attention and reaction time as well as in quality of care during acute exposure to altitude.

**Objective:**

The primary aim of the current study was to investigate the effect of oxygen supplementation on cognitive performance in Helicopter EMS (HEMS) providers during acute exposure to altitude.

**Design, setting, and participants:**

This interventional, randomized, controlled, double-blind, cross-over clinical trial was conducted in October 2021. Each trial used a simulated altitude scenario equivalent to 4000 m, in which volunteers were exposed to hypobaric hypoxia with a constant rate of ascent of 4 m/s in an environmental chamber under controlled, replicable, and safe conditions. Trials could be voluntarily terminated at any time. Inclusion criteria were being members of emergency medical services and search and rescue services with an age between 18 and 60 years and an American Society of Anesthesiologists physical status class I.

**Exposures:**

Each participant conducted 2 trials, one in which they were exposed to altitude with oxygen supplementation (intervention trial) and the other in which they were exposed to altitude with ambient air supplementation (control trial).

**Main outcomes and measures:**

Measurements included peripheral oxygen saturation (SpO_2_), cerebral oxygenation (ScO_2_), breathing and heart rates, Psychomotor Vigilance Test (PVT), Digit-Symbol Substitution Test (DSST), *n*-Back test (2-BACK), the Grooved Pegboard test, and questionnaires on subjective performance, stress, workload, and positive and negative affect. Paired t-tests were used to compare conditions (intervention vs. control). Data were further analyzed using generalized estimating equations (GEE).

**Results:**

A total of 36 volunteers (30 men; mean [SD] age, 36 [9] years; mean [SD] education, 17 [4] years) were exposed to the intervention and control trials. The intervention trials, compared with the control trials, had higher values of SpO_2_ (mean [SD], 97.9 [1.6] % vs. 86 [2.3] %, t-test, *p* = 0.004) and ScO_2_ (mean [SD], 69.9 [5.8] % vs. 62.1 [5.2] %, paired t-test, *p* = 0.004). The intervention trials compared with the control trials had a shorter reaction time (RT) on the PVT after 5 min (mean [SD], 277.8 [16.7] ms vs. 282.5 [15.3] ms, paired t-test, *p* = 0.006) and after 30 min (mean [SD], 276.9 [17.7] ms vs. 280.7 [15.0] ms, paired t-test, *p* = 0.054) at altitude. While controlling for other variables, there was a RT increase of 0.37 ms for each % of SpO_2_ decrease. The intervention trials showed significantly higher values for DSST number of correct responses (with a difference of mean [SD], 1.2 [3.2], paired t-test, *p* = 0.035). Variables in the intervention trials were otherwise similar to those in the control trials for DSST number of incorrect responses, 2-BACK, and the Grooved Pegboard test.

**Conclusions and relevance:**

This randomized clinical trial found that oxygen supplementation improves cognitive performance among HEMS providers during acute exposure to 4000 m altitude. The use of oxygen supplementation may allow to maintain attention and timely reaction in HEMS providers. The impact of repeated altitude ascents on the same day, sleep-deprivation, and additional stressors should be investigated.

*Trial registration* NCT05073406, ClinicalTrials.gov trial registration.

## Introduction

### Background

At high altitude (HA) there is lower barometric pressure and a lower partial pressure of oxygen in ambient air than at low altitude [1]. Emergency medical services (EMS) providers transiently ascend to HA for primary missions and secondary transports in mountainous areas in helicopters that are unpressurised and do not have facilities for oxygen supplementation [2]. They must provide medical treatment during potentially complex technical operations. In two randomized, controlled, single-blind, crossover trials, 48 providers active in Helicopter Emergency Medical Service (HEMS) showed impairments of attention and reaction time (RT), and of quality of care during simulated HA scenario [3, 4]. The impairments were correlated with the decreases in oxygen saturation. Other studies have shown cognitive impairment at altitudes above 3000 m with increases in procedural errors [5] and declines in working memory, and executive function or abstract reasoning [6, 7] in high-performance providers.

### Importance

Crashes are one of the greatest hazards faced in both ambulance EMS [8] and HEMS missions [9, 10]. Reduced cognitive performance during HEMS missions at altitude could lead to accidents and to decreased quality of care. HEMS providers do not appear to be aware of the reduced performance during HA exposure [3, 4]. To our knowledge, no data have been reported on the efficacy of oxygen supplementation to prevent cognitive impairment in HEMS providers at altitude.

### Goals of this investigation

The primary aim of the current study was to investigate the effect of oxygen supplementation on cognitive performance in HEMS providers during acute exposure to HA. We studied selected cognitive domains, including attention, working memory, psychomotor speed, fine motor skills, and visuomotor tracking. Secondary aims of the proposed study were to investigate subjective assessment of cognitive performance, mental stress, workload, and experienced positive and negative affect.

## Methods

This interventional, randomized, controlled, double-blind, cross-over clinical trial was approved by the institutional review board of Bolzano (Protocol Number 0228969-BZ) and registered in ClinicalTrials.gov (Protocol Record NCT05073406). We conducted the study in adherence to the Declaration of Helsinki. All participants were informed about the possible risks of being exposed to altitude and gave written informed consent prior to enrollment. This study is reported following the Consolidated Standards Of Reporting Trials (CONSORT) reporting guideline.

### Study population

The participants were healthy, unpaid, providers of EMS and search and rescue (SAR) services with occupational licenses. Participants of both sexes, with age between 18 and 60 years old and classified according to the American Society of Anesthesiologists (ASA) as class I were considered eligible [11]. Exclusion criteria were age below 18 years, age above 60 years, ASA class greater than I, a medical history of psychiatric disorders and neurological diseases, or severe altitude illness [12], and any acute disease. Participants were asked to avoid sleep deprivation and abstain from caffeine, alcohol consumption and smoking prior to the trials.

### Randomization

A randomization list was created with the use of computer-generated pseudo-random numbers, balanced for sequence (intervention then control or control then intervention) and daytime (morning, afternoon). The sequence of cognition tests was randomized and balanced within each test session. Participants and researchers were blinded toward the oxygen supplementation.

### Study protocol

**Fig. 1:**
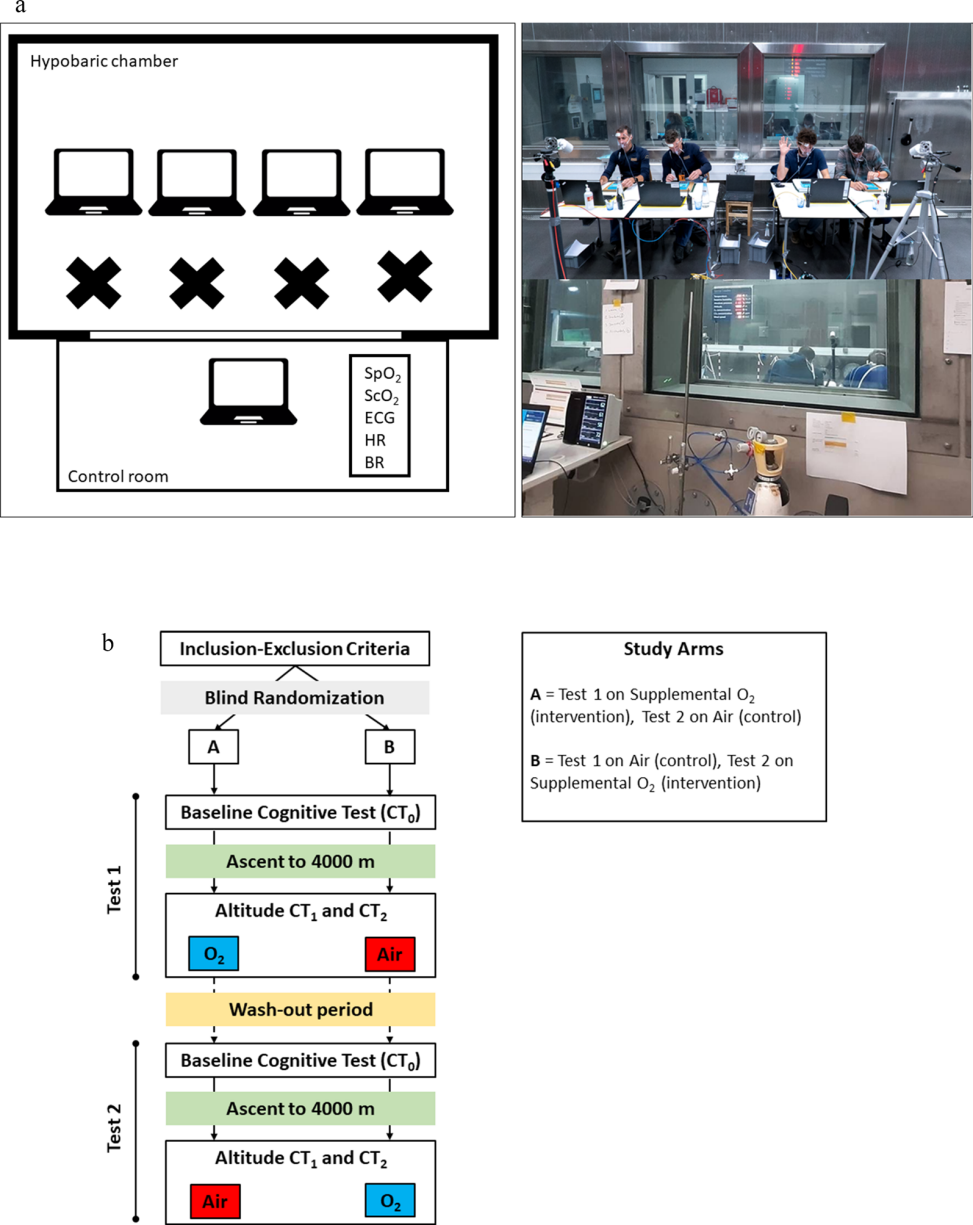
Experimental set-up. Panel **a.** Schematic representation of the study setting in the *terraXcube* in Bolzano, Italy, (on the left) for each study arm (4 participants) inside of the hypobaric chamber (upper panel) and of the control room (lower panel). Pictures (on the right) refer to the experimental setup inside the hypobaric chamber (upper panel) and in the control room (lower panel). Panel** b**. Study design. BR, breath rate; CT, cognitive tests; HR, heart rate; ScO_2_, cerebral oxygenation saturation; SpO_2_, peripheral oxygen saturation

Each participant performed 2 trials (intervention and control) in a crossover design on the same day. Participants were divided into 9 groups of 4 participants each and the crossover design consisted of 2 study arms as shown in Fig. 1.

In the intervention trial, each participant was exposed to altitude with continuous oxygen supplementation of 2 l/min via OxyMask (Southmedic Inc., Barrie, ON, Canada). In the placebo control trial, each participant was exposed to altitude with continuous ambient air supplementation of 2 l/min via OxyMask.

Prior to the study, participants completed a medical interview with a general medical visit (including self-assessments for sleep quality, depression/anxiety, perceived mental stress, and altitude exposure). Cognitive tests were administered three times during each trial: before the ascent (CT_0_), at 5 min (CT_1_) and 30 min (CT_2_) after the end of the ascent. After completing each cognitive test session, participants self-rated their cognitive performance and stress perception, as well as the positive and negative affect, and then performed the Grooved Pegboard test [13] twice, once with the dominant hand and once time with the non-dominant hand. At the end of each trial, participants rated their perceived workload.

Prior to the trial, participants rested for approximately 20–30 min to minimize anxiety and stress and performed a familiarization session of the cognitive tests and the Grooved Pegboard test. Trials could be voluntarily terminated at any time.

### Setting and instrumentation

All trials were performed in the environmental chamber *terraXcube*, Eurac Research, Bolzano, Italy in October 2021 (Fig. 1). Experiments were conducted at a simulated altitude scenario equivalent to 4000 m, with volunteers exposed to hypobaric hypoxia at a constant rate of ascent of 4 m/s. *terraXcube* temperature, humidity, and carbon dioxide were continuously monitored and kept constant at normal indoor values.

Oxygen and air supplementation were delivered through OxyMask, designed to concentrate and redirect the flow of oxygen/air, preventing carbon dioxide re-breathing.

Belts for the monitoring system (Equivital EQ02, Hidalgo, UK) and sensors for oxygen saturation were fitted and participants received a technical introduction and safety briefing for *terraXcube.*

Cognitive tests were performed on dedicated laptops, which were placed on a separate desk for each participant. Participants were continuously monitored and guided (via radio commands) from the control room of *terraXcube* by research staff.

### Measurements

Sustained attention was assessed with the Psychomotor Vigilance Test (PVT), visual attention and psychomotor speed with the Digit Symbol Substitution Test (DSST), and working memory with the 2-back Numerical (2-BACK) task. A brief 3-min version of the PVT was used, based on simple RT to visual stimuli, that occurred at random intervals varying from 2 to 5 s in steps of 200 ms, as previously described [3, 14–17]. In the PVT, RT (milliseconds [ms]) (excluding lapses and false starts), number of omission errors or “lapses” (defined as RTs  ≥ 355 ms), false starts or errors of commission (defined as a response with no stimulus or RT < 100 ms), and performance score (defined as 1 minus the number of lapses and false starts divided by the number of valid stimuli including false starts ranging from 0 to 100%) were evaluated [15]. A computerised version of the DSST with 9 specific nonsense symbols was used, as previously described [3, 14, 18]. Test duration was fixed at 90 s, and the legend key was randomly reassigned at each administration. In the DSST, mean total number of correct and incorrect response pairs were evaluated. A 2-back Numerical (2-BACK) task of the *n*-back Numerical test was used [19]. Participants had to identify and indicate if the item currently presented was the same as the item presented 2 items earlier. In the 2-BACK, number of correct responses, number of missed responses, number of incorrect responses, mean RT of both correct and incorrect responses were evaluated. The software containing the three cognitive tests was installed on dedicated laptops as previously described [3, 14]. Six different versions of the DSST and the 2-BACK were administered across the multiple time points (test 1 and test 2, and CT_0_, CT_1_ and CT_2_) to avoid learning effects. The three tests were randomly assigned.

Fine motor skills, visuomotor tracking, and response speed (including motor inhibition and cognitive flexibility) were assessed with the Grooved Pegboard (Lafayette Instrument, Lafayette, IN, USA) [13]. Participants inserted 25 pegs into the grooved slots in a standardized order and as quickly as possible.

Perceived mental stress, anxiety and depression, and sleep quality were evaluated prior to the initiation of the study using the Perceived Stress Scale (PSS)-10 item [20], the Hospital Anxiety (HADS-A) and Depression (HADS-D) Scale [21], the Pittsburgh Sleep Quality Index (PSQI) [22], and the Insomnia Severity Index (ISI) [23]. Subjective performance and mental stress perception following each cognitive test session were evaluated using a visual analogue scale (VAS) [24]. Participants placed a mark on a 100-mm VAS horizontally positioned with the extremes labelled very good-very bad and low stress-high stress. Positive and negative affect were evaluated by the Positive and Negative Affect Schedule (PANAS) scale [25]. Participants rated their perceived workload using the National Aeronautics and Space Administration Task Load Index (NASA-TLX) questionnaire [26].

Physiological parameters measured continuously and noninvasively included heart rate (HR), derived from the 2-lead electrocardiogram, breathing rate (BR), SpO_2_ by a forehead sensor (EQ02, Hidalgo, UK), and ScO_2_ by near-infrared spectroscopy (NIRS), also by a forehead sensor (O3 Regional Oximetry, Masimo). The NIRS sensors were placed at a standardized frontotemporal location, high on the forehead to avoid any influences from the frontal or sagittal sinuses. The SpO_2_ sensor on the forehead was applied on the opposite side to the NIRS.

### Statistical analysis

To achieve a power of 80% with *p* < 0.05, we estimated that we would need a sample size of 36 participants for an effect size of 0.67 between the oxygen supplementation tests and the control tests. For RT the clinically significant mean difference was 10 ± 15 ms [27].

We used SPSS for Windows software version 29.0 (SPSS, Chicago, IL, United States) to build the database and for statistical analysis. We used PRISM 10 (GraphPad Software) for visualizations. Continuous variables were expressed as mean and standard deviation (SD), whereas percentages were used for counted data. Paired t-tests were used to compare conditions (intervention vs. control). Data were further analyzed using generalized estimating equations (GEE) with condition and session (at 5 min and 30 min) as a within effect and using a first-order autoregressive (AR) [28] working correlation matrix. Predictors were condition (intervention, control), daytime (morning, afternoon), session, the interaction between daytime and session, gender, age above 33 years, education above 13 years, ISI score above 7, and the overall baseline (mean of morning and afternoon baseline measurements) as covariates. Instead of the condition effect, SpO_2_ at each timepoint was included in the model as a covariate. For graphical presentation, a heatmap was calculated showing the different means per session and variable for the conditions (intervention vs. control) [28]. For each variable z-scores were calculated and oriented so that higher values corresponded to better values. Colors were then assigned to all mean values according to percentiles.

We considered a two-sided *p*-value below 0.05 to be significant. We used a two-step rejection procedure to account for multiple hypothesis testing and to adjust *p*-values for a single predictor [29].

## Results

**Fig. 2:**
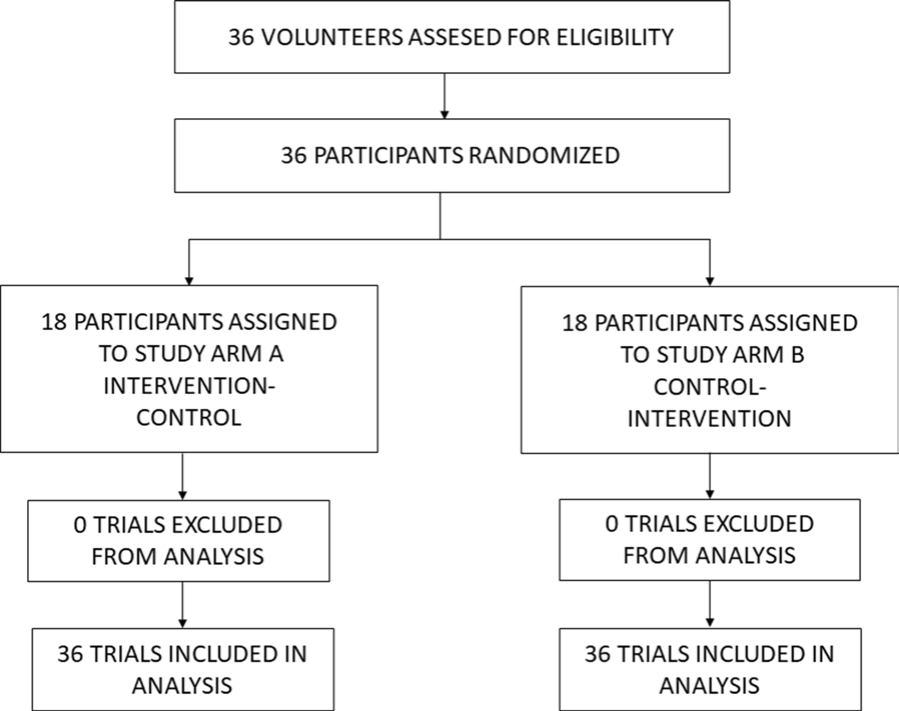
Participant Flowchart

Thirty-six volunteers were assessed for eligibility and enrolled to participate in the study (Fig. 2). All of them (30 men; mean [SD] age, 36 [8] years; mean [SD] education, 17 [4] years; 36 right-hand dominant) participated in both the intervention and control trials and were included in the data analysis. Characteristics of participants are reported in Table 1. Mean [SD] PSS score was 10.4 [5.4]. Mean [SD] ISI score was 3.7 [3.5]. Mean [SD] score of HADS-A was 2.9 [2.1] and HADS-D was 1.8 [1.6]. Mean [SD] PSQI score was 3.8 [1.8].

**Table 1:** Demographic data and baseline questionnaires (36 participants)

Parameters	Values
Males, no. (%)	30 (83%)
Age, mean ± SD, y	36.4 ± 8.5
Education, mean ± SD, y	16.7 ± 3.6
PSS mean ± SD, cut off ≥ 14, No. (%)	10.4 ± 5.4, 11 (31)
ISI mean ± SD, cut-off > 7, No. (%)	3.7 ± 3.5, 5 (14)
HADS-A mean ± SD, cut-off ≥ 8, No. (%)	2.9 ± 2.1, 0 (0)
HADS-D mean ± SD, cut-off ≥ 8, No. (%)	1.8 ± 1.6, 0 (0)
PSQI mean ± SD, cut-off > 5, No. (%)	3.8 ± 1.8, 6 (36)
VAS stress (range 0–100)	30.1 ± 19.2
VAS performance (range 0–100)	42.4 ± 15.2
PANAS-P (range 10–50)	31.9 ± 6
PANAS-N (range 10–50)	11.9 ± 1.8
Grooved Pegboard test, dominant hand (s)	58.3 ± 6.8

HADS, Hospital Anxiety Depression Scale; ISI, Insomnia Severity Index; PANAS, Positive (P) and Negative (N) Affect Schedule; PSQI, Pittsburgh Sleep Quality Index; PSS, Perceived Stress Scale; VAS, Visual Analogue Scale of subjective performance and stress rating

**Fig. 3:**
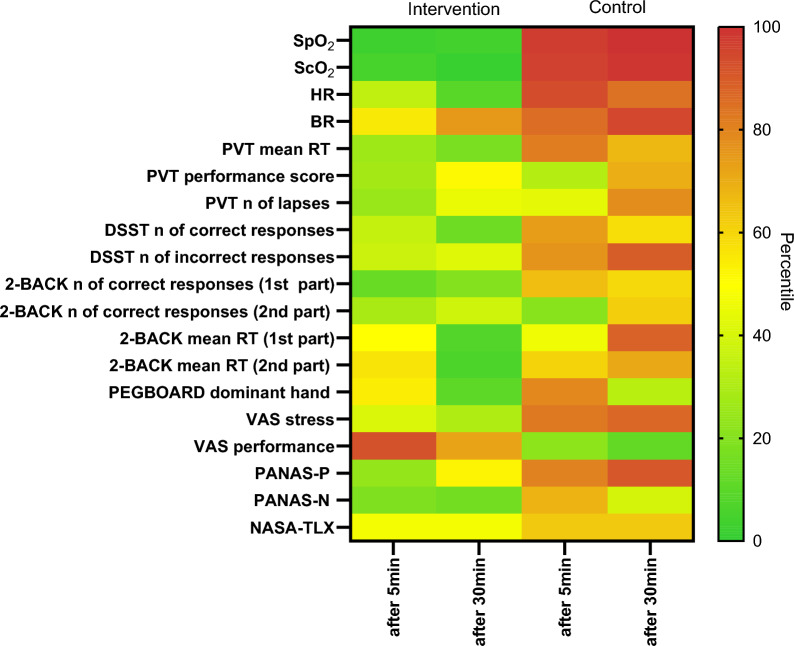
Heatmap representing the different means per session and variable for intervention (with O_2_) and control (without O_2_) conditions. For every variable z-scores were calculated and oriented such that higher values (green vs. red) correspond to better values. Colors were then assigned to all mean values according to percentiles. 2-BACK, 2-back Numerical task; BR, breath rate; DSST, Digit Symbol Substitution Test; HR, heart rate; mean, mean of values after 5 and 30 min; NASA-TLX, NASA Task Load Index; PANAS, Positive (P) and Negative (N) Affect Schedule; PEGBOARD, Grooved Pegboard test; PVT, Psychomotor Vigilance Test; RT, reaction time; ScO_2_, cerebral oxygenation saturation; SpO_2_, peripheral oxygen saturation; VAS, Visual Analogue Scale

The heatmap shows the relatively different effects of oxygen supplementation in the intervention trials compared with the control trials on the parameters evaluated, with the green squares showing an improvement and the red squares showing a worsening (Fig. 3).

**Fig. 4:**
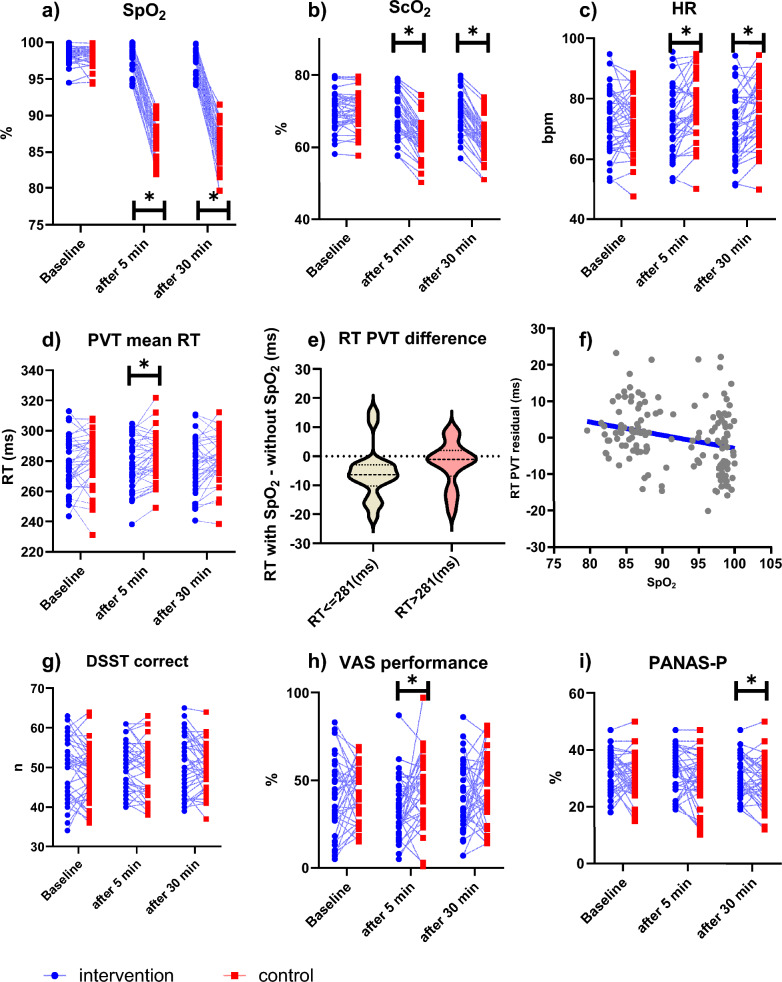
Cognitive tests, physiological responses, performance perception and positive affect between intervention and control trials. Individual data is shown for intervention (blue) and control (red) for SpO_2_ (a), ScO_2_ (**b**), HR (**c**), PVT mean RT (**d**), DSST number of correct responses (**g**, VAS performance (**h**), and PANAS-P (**i**). Measurements of the same participant are connected by lines. Violin plots with 95% confidence intervals for the mean are shown in (**e**) for the within-person difference in reaction time in PVT separately for participants who had a baseline reaction time below or above 281 ms. A scatter plot with the respective regression line between SpO_2_ and the RT residual of the GEE model, when correcting for daytime, sequence, sex, age, ISI, HR, BR, and baseline RT, is shown in (**f**)

The intervention trials compared with the control trials had higher values of mean SpO_2_ (mean [SD], 97.9 [1.6] % vs. 86.0 [2.3] %, paired t-test, *p* = 0.004) and mean ScO_2_ (mean [SD], 69.9 [5.8] % vs. 62.1 [5.2] %, paired t-test, *p* = 0.004) and comparable values for mean BR (mean [SD], 16.1 [2.9] bpm vs. 15.3 [3.0] bpm, paired t-test, *p* = 0.288). The intervention trials compared with the control trials had lower values of mean HR (mean [SD], 71.6 [10.7] bpm vs. 76.5 [10.1] bpm, paired t-test, *p* = 0.003) (Table 2 and Fig. 4 panel a,b,c).

**Table 2:** Paired t-test of comparison between intervention and control trials

Parameter	Session	n	Intervention		Control		Corrected *p*-value
			Mean	SD	Mean	SD	paired t-test
SpO_2_ (%)	Baseline	36	98.2	1.2	98.2	1.2	0.992
	After 5 min	36	98.0	1.6	86.4	2.2	**0.004**
	After 30 min	36	97.8	1.5	85.6	2.6	**0.004**
	Mean	36	97.9	1.6	86.0	2.3	**0.004**
ScO_2_ (%)	Baseline	36	70.0	5.1	70.1	5.1	0.962
	After 5 min	35	69.6	5.9	62.4	5.5	**0.004**
	After 30 min	34	69.8	5.6	61.4	4.9	**0.004**
	Mean	35	69.9	5.8	62.1	5.2	**0.004**
BR (bpm)^a^	Baseline	36	17.3	3.0	17.3	3.0	0.950
	After 5 min	36	16.3	3.1	15.7	3.3	0.815
	After 30 min	36	15.9	2.9	15.0	2.9	0.116
	Mean	36	16.1	2.9	15.3	3.0	0.288
HR (bpm)^b^	Baseline	36	72.7	10.8	72.2	9.3	0.700
	After 5 min	36	72.7	10.8	77.6	10.3	**0.003**
	After 30 min	36	70.6	10.8	75.4	10.0	**0.003**
	Mean	36	71.6	10.7	76.5	10.1	**0.003**
PVT mean RT (ms)	Baseline	36	279.4	17.6	282.0	17.6	0.230
	After 5 min	36	277.8	16.7	282.5	15.3	**0.006**
	After 30 min	36	276.9	17.7	280.7	15.0	0.054
	Mean	36	277.3	16.7	281.6	14.5	**0.005**
PVT performance score	Baseline	36	0.9	0.1	0.9	0.1	0.886
	After 5 min	36	0.9	0.1	0.9	0.1	0.897
	After 30 min	36	0.9	0.1	0.9	0.1	0.845
	Mean	36	0.9	0.1	0.9	0.1	0.867
PVT n of lapses	baseline	36	2.8	3.3	3.0	2.8	0.601
	After 5 min	36	2.2	3.1	2.6	4.7	0.556
	After 30 min	36	2.6	3.5	3.1	3.6	0.430
	Mean	36	2.4	3.1	2.9	3.9	0.413
DSST n of correct responses	Baseline	36	49.8	7.6	48.8	6.8	0.234
	After 5 min	36	50.4	6.1	49.3	6.6	0.096
	After 30 min	36	51.3	7.3	49.9	5.8	0.125
	Mean	36	50.8	6.5	49.6	5.8	**0.035**
DSST n of incorrect responses	Baseline	36	0.5	0.7	0.8	1.3	0.094
	after 5 min	36	0.7	1.1	0.9	15	0.401
	After 30 min	36	0.8	0.9	1.1	1.7	0.289
	Mean	36	0.7	0.8	1.0	1.2	0.152
2-BACK n of correct responses	Baseline	36	21.1	3.0	21.2	2.5	0.885
	After 5 min	32	21.7	3.1	21.7	2.2	0.924
	After 30 min	34	22.0	2.6	21.7	2.5	0.858
	Mean	35	21.8	2.6	21.7	2.1	0.894
2-BACK n of correct responses (1st part)	Baseline	35	10.5	2.6	10.1	2.5	0.535
	After 5 min	34	11.2	2.3	10.5	2.6	0.344
	After 30 min	33	11.1	2.3	10.7	2.4	0.518
	Mean	36	11.1	1.6	10.7	1.7	0.330
2-BACK n of correct responses (2nd part)	Baseline	35	10.3	2.9	10.6	2.3	0.810
	After 5 min	34	11.1	2.3	11.3	2.5	0.851
	After 30 min	33	10.9	2.1	10.8	2.8	0.875
	Mean	36	11.1	1.5	11.0	1.8	0.861
2-BACK n of missed responses	Baseline	36	2.9	3.0	2.8	2.5	0.585
	After 5 min	36	4.2	6.7	2.9	4.2	0.420
	After 30 min	36	3.3	5.7	2.4	2.5	0.451
	Mean	36	3.7	5.2	2.6	2.9	0.335
2-BACK n of incorrect responses	Baseline	36	1.3	1.8	1.3	1.1	0.833
	After 5 min	36	1.1	1.3	1.3	1.4	0.719
	After 30 min	36	1.5	2.0	1.1	1.3	0.669
	Mean	36	1.3	1.4	1.2	1.0	0.821
2-BACK correct RT mean (ms)	Baseline	36	0.6	0.2	0.6	0.2	0.976
	After 5 min	32	0.6	0.2	0.6	0.2	0.972
	After 30 min	34	0.6	0.2	0.6	0.2	0.966
	Mean	35	0.6	0.2	06	0.2	0.984
2-BACK correct mean RT (1st part) (ms)	Baseline	35	0.7	0.2	0.6	0.2	0.380
	After 5 min	34	0.6	0.2	0.6	0.2	0.980
	After 30 min	33	0.5	0.1	0.6	0.3	0.236
	Mean	36	0.5	0.1	0.6	0.1	0.376
2-BACK correct mean RT (2nd part) (ms)	Baseline	35	0.7	0.2	0.6	0.2	0.841
	After 5 min	34	0.6	0.2	0.6	0.2	0.928
	After 30 min	33	0.5	0.1	0.6	0.2	0.232
	Mean	36	0.5	0.1	0.6	0.1	0.464
2-BACK incorrect RT mean (ms)	Baseline	17	0.8	0.4	0.8	0.5	0.778
	After 5 min	15	0.8	0.3	0.8	0.4	0.709
	After 30 min	13	0.7	0.4	0.6	0.2	0.556
	Mean	25	0.7	0.3	0.7	0.3	0.790
PEGBOARD dominant hand (s)	Baseline	36	57.6	7.5	59.0	8.5	0.317
	After 5 min	36	57.1	6.1	58.3	9.6	0.342
	After 30 min	36	55.1	6.9	56.5	8.0	0.216
	Mean	36	56.1	6.0	57.4	8.2	0.201
VAS stress (range 0–100)	Baseline	36	26.4	18.7	33.8	25.0	0.059
	After 5 min	36	31.2	23.1	36.4	22.4	0.193
	After 30 min	36	30.0	22.9	36.9	24.3	0.062
	Mean	36	30.6	21.1	36.7	21.9	0.069
VAS performance (range 0–100)	Baseline	36	40.8	21.6	43.9	15.3	0.405
	After 5 min	36	34.8	17.0	44.7	19.7	**0.021**
	After 30 min	36	40.0	19.5	46.0	20.3	0.221
	Mean	36	37.4	17.0	45.3	18.6	0.060
PANAS-P (range 10–50)	Baseline	36	32.9	6.9	30.8	7.3	0.110
	After 5 min	36	31.9	8.0	29.7	9.0	0.141
	After 30 min	36	31.0	7.0	28.4	8.1	**0.028**
	Mean	36	31.4	7.3	29.1	8.2	0.063
PANAS-N (range 10–50)	Baseline	36	12.2	2.9	11.6	2.0	0.394
	After 5 min	36	11.2	1.3	11.7	2.0	0.238
	After 30 min	36	11.1	2.1	11.4	2.0	0.504
	Mean	36	11.2	1.6	11.5	1.8	0.323
NASA-TLX	Baseline	36	57.7	14.8	58.5	14.4	0.554
	After 5 min	36	57.7	14.8	58.5	14.4	0.554
	After 30 min	36	57.7	14.8	58.5	14.4	0.554
	Mean	36	57.7	14.8	58.5	14.4	0.554

Continuous variables are expressed as mean and standard deviation

*p*-values are given for each covariate effect on each variable. *p*-values have been corrected for multiple variable comparisons. *p*-values < 0.05 are in bold

2-BACK, 2-back Numerical task; bpm^a^, beat per minute; bpm^b^, breaths per minute; BR, breath rate; DSST, Digit Symbol Substitution Test; HR, heart rate; Mean, mean of values after 5 and 30 min; n, number; NASA-TLX, NASA Task Load Index; O_2_, oxygen; PANAS, Positive (P) and Negative (N) Affect Schedule; PEGBOARD, Grooved Pegboard test; PVT, Psychomotor Vigilance Test; RT, reaction time; ScO_2_, cerebral oxygenation saturation; SD, standard deviation; SpO_2_, peripheral oxygen saturation; VAS, Visual Analogue Scale of subjective performance and stress

### Primary outcomes

The intervention trials compared with the control trials had shorter RT on the PVT (mean [SD], 277.8 [16.7] ms vs. 282.5 [15.3] ms, paired t-test, *p* = 0.006) at 5 min and (mean [SD], 276.9 [17.7] ms vs. 280.7 [15.0] ms) paired t-test, *p* = 0.054) at 30 min as well as at the mean (mean of 5 and 30 min) (mean [SD], 277.3 [16.7] ms vs. 281.6 [14.5] ms, paired t-test, *p* = 0.005) (Table 2 and Fig. 4 panel d). The intervention trials compared with the control trials had similar performance scores and number of lapses on the PVT. The intervention trials compared with the control trials had a higher mean (mean of 5 and 30 min) number of correct responses (>mean [SD], 50.8 [6.5] vs. 49.6 [5.8], paired t- test, *p* = 0.035) and a similar number of incorrect responses on the DSST (Table 2 and Fig. 4 panel g). The intervention trials compared with the control trials had similar number of correct, missed, and incorrect responses, as well as the mean RT of both correct and incorrect responses on the 2-BACK (Table 2). The intervention trials compared with the control trials had shorter but not statistically significant different RTs in both the first part (mean [SD], 0.5 [0.1] ms vs. 0.6 [0.1] ms, paired t-test not corrected, *p* = 0.059) and the second part (mean [SD], 0.5 [0.1] ms vs. 0.6 [0.1] ms, paired t- test not corrected, *p* = 0.058) at 30 min. The intervention trials compared with the control trials had similar time to insert the pegs in the pegboard at the Grooved Pegboard test with the dominant hand (Table 2).

### Secondary outcomes

The intervention trials compared with the control trials had lower but not statistically significant different mean VAS stress scores (mean [SD], 30.6 [21.1] mm vs. 36.7 [21.9] mm, paired t-test, *p* = 0.069), and mean VAS performance scores (mean [SD], (37.4 [17] mm vs. 45.3 [18.6] mm, paired t-test, *p* = 0.060) (Table 2 and Fig. 4 panel h). The intervention trials compared with the control trials had higher but not statistically significant different mean PANAS-P scores (mean [SD], 31.4 [7.3] vs. 29.1 [8.2]), paired t-test, *p* = 0.063 (Table 2 and Fig. 4 panel i). The intervention trials compared with the control trials had similar mean PANAS-N scores. The intervention trials compared with the control trials had similar mean NASA-TLX scores.

### Other analyses for the primary outcomes

GEE analysis confirmed an independent effect of condition (intervention vs. control trials) on RT on the PVT (GEE, *p* = 0.017). GEE analysis also showed an independent influence of ISI on RT on the PVT (GEE, *p* = 0.026) (Table 3). Without condition effect, SpO_2_ showed a significant effect on RT on the PVT (GEE beta − 0.37, *p* < 0.001). Thus, for each % decrease in SpO_2_, there was an increase in RT of 0.37 ms (Fig. 4 panel f). Volunteers who were faster at baseline (RT ≤ 281 ms) showed a greater slowing of RT compared to those who were slower at baseline (RT > 281 ms) (mean difference in RT [SD] − 0.9 [8.9] ms, one sample t-test, *p* = 0.011 vs. − 2.6 [7.7] ms, one sample t-test, *p* = 0.171) (Fig. 4 panel e).

**Table 3:** *p*-values of effects estimated by generalized estimating equations (GEE)

Test	Parameter	Condition	Day time	Session	Day time* Session	Gender	Age	Education	ISI	Overall baseline
PVT	Mean RT (≥ 355 ms)	**0.017**	0.714	0.681	**0.017**	0.485	0.917	0.884	**0.026**	** < 0.001**
	Performance score	**0.904**	0.837	0.686	0.854	0.002	0.096	0.337	0.192	** < 0.001**
	Number of lapses	0.694	0.867	0.704	0.710	0.164	0.662	0.229	0.623	** < 0.001**
DSST	N of correct responses	**0.139**	0.363	0.270	0.773	0.120	0.688	0.886	0.210	** < 0.001**
	N of incorrect responses	0.527	0.778	0.889	0.736	0.194	0.669	0.190	0.589	** < 0.001**
2-BACK	N of correct responses (1st part)	0.618	0.344	0.921	0.630	0.431	0.888	0.589	0.780	0.214
	N of correct responses (2nd part)	0.890	0.679	0.840	0.713	**0.032**	0.893	**0.007**	0.254	** < 0.001**
	Mean RT 1st part	0.458	0.659	0.880	0.707	**0.000**	0.708	0.741	0.745	0.265
	Mean RT 2nd part	0.333	0.212	0.737	0.759	0.494	0.886	0.864	0.630	0.161
PEGBOARD	Dominant hand	0.506	**0.001**	0.116	0.061	0.470	**0.029**	0.821	0.516	** < 0.001**

An asterisk (*) placed between two factors indicates the interaction effect of those two factors. 2-BACK, 2-back Numerical task; DSST, Digit Symbol Substitution Test; ISI, Insomnia Severity Index; PEGBOARD, Grooved Pegboard test; PVT, Psychomotor Vigilance Test; RT, Reaction Time

GEE analysis showed no independent influence of condition (intervention vs. control trials) on performance score (GEE, *p* = 0.904) and the number of lapses on the PVT (GEE, *p* = 0.694), and on the parameters analyzed on the DSST, 2-BACK and the Grooved Pegboard test (Table 3).

## Discussion

This randomized clinical trial found that oxygen supplementation improves cognitive performance in HEMS providers during acute exposure to an altitude equivalent to 4000 m. The exposure in the control trial induced reduced sustained attention and timely reactions (i.e., slowing of RT). The increase in RT was inversely correlated to the decrease in oxygen saturation. Oxygen supplementation blunted the decrease in sustained attention and slowing of RT seen in the control trials. This suggests that the use of oxygen supplementation may be an effective countermeasure to improve occupational safety and health in providers of HEMS services operating at high altitude.

Cognitive impairment at HA is well known in aviation both in military and commercial contexts [30]. The rate and the length of hypoxia exposure in aviation compared to helicopter operations is substantially different. Previous studies demonstrated cognitive impairment at altitudes above 3000 m [5, 6, 31–34]. Nesthus et al. [5] reported more procedural errors during simulated flights in 20 private pilots at 3048 m and 3810 m. Bouak et al. [6] reported a decline in cognitive performance (i.e., short-term and working memory, executive function) at 4267 m and a lower positive mood (assessed with PANAS) in 16 military helicopter pilots. Pilmanis et al. [31] showed a minimal negative effect of simulated hypobaric hypoxia at 3658 m on cognitive performance (working memory and abstract reasoning) in 91 participants from military personnel. Peacock et al. [32] showed executive functioning impairment in 10 pilots at the simulated altitude of 3810 m with no effect on flight performance. Steinman et al. [33–35] showed that in helicopter crews exposed to a simulated altitude of 4572 m there was reduced alertness and awareness of the environment, decreased flight performance and increased RT [33–35] while there was no significant effect on flight performance at 3048 m [35]. We previously showed that acute exposure to an altitude of 5000 m of HEMS providers resulted in a slowing of RT that was not subjectively perceived, while psychomotor speed and decision making were not affected [3]. We found a decrease in sustained attention and lengthening of reaction times at 4000 m in EMS and SAR providers but no significant impairment of working memory, fine motor skills, visuomotor tracking and psychomotor speed. Increased reaction times were inversely correlated with the decrease in oxygen saturation, confirming previous findings that cognitive impairments at simulated altitude below 4000 m are insignificant [3, 7, 31].

There is an individual susceptibility and variability in responding to hypoxia (e.g., extent of the hypoxic ventilatory response, increased parasympathetic or sympathetic activity). Previous studies in aviation reported hypoxia symptoms in some individuals even at low altitudes [36, 37]. We found individual differences also in HEMS providers. There was a range of reaction times and the participants with faster reaction times at baseline (273.9 ms) had relatively greater lengthening of RTs in hypoxia without supplemental oxygen compared to the slower (289.4 ms) ones, but we did not find any influence of age or gender.

We found that oxygen supplementation improved the reduced sustained attention and timely reactions (i.e., slowing of RT) in HEMS providers during acute exposure to altitude. There was a positive correlation between cognitive performance and oxygenation level. Oxygen was administered at 2 l/min reaching a peripheral oxygen saturation of around 95–100% without administering an excess of oxygen and avoiding any carbon dioxide re-breathing through the use of an open design mask [38].

Our results are important for an evidence-based development of occupational safety regulation of providers operating in HEMS. Helicopters (e.g., H145, Airbus Helicopter SAS, Marignane, France; AW139, Leonardo, Cascina Costa di Samarate (VA), Italy; Bell 429, Bell, Fort Worth, TX, USA) operating in EMS transiently ascend to high altitude for primary missions and secondary transport in areas such as Europe, Colorado and Alaska in the USA [39], as well as in countries that have high mountain ranges in Asia and South America [40–42]. Helicopters do not need to fly at HA in the cruise phase unlike airplanes. Based on this assumption, the European Aviation Safety Agency stated that the need for oxygen is lower in HEMS mountain rescue operations because of the shorter time periods spent at altitude compared to general aviation [43]. Our results suggest a potential benefit in the use of oxygen supplementation for missions and transport at altitudes ≥ 4000 m also in HEMS operations. Reduced attention and increased reaction time can be observed during a single ascent of a HEMS mission [3, 44]. Aviation safety agencies and HEMS should consider oxygen supplementation based on the altitude, the time of exposure, the procedures [4, 45], as well as the impact of additional stressors. Multiple exposures to HA in daily practice can have an impact on cognition as reported by Robinson et al. [46] who reported flight performance deterioration during exposure to simulated 10,000 ft preceded by exposure to 25,000 ft. Some flight phases, such as take-off and landing, have been associated with increased number of HEMS accidents [10]. Sleep deprivation can be an important additional stressor. We found and independent effect of ISI on RT. A similar slowing of RT was reported in another population of health-care providers (i.e., nurses) due to sleep deprivation after night shift compared to day shift [47]. A lengthening of RT and an increase in self-reported tiredness was found for HEMS crew over a 5-week shift cycle [48].

Our results also showed that supplemental oxygen reduced the subjective mental stress level (as measured by the VAS) and increased the propensity for positive emotions (PANAS-P) thereby possibly affecting the management of other challenging situations.

## Limitations

This was a simulation study. The results may not apply to real-world situations. The experimental protocol involved two consecutive ascents on a single day. In practice HEMS personnel may participate in multiple missions daily for multiple days, possibly causing larger decreases in cognitive performance. The clinical trial was run under controlled, replicable, and safe conditions that did not allow evaluation of the efficacy of oxygen supplementation in participants who experienced additional processive and systemic stressors. The computerized PVT lacks normative data taking into account different age and sex, as well as performance. The experimental protocol controlled the effect of sleep deprivation but it did not investigate the effect of multiple altitude ascents on the same day and other additional stressors.

## Conclusions

This randomized clinical trial found that oxygen supplementation improves cognitive performance in HEMS providers during acute exposure to altitude. The use of oxygen supplementation may allow to maintain sustained attention and timely reactions in HEMS providers. The impact of repeated altitude ascents on the same day, weather conditions, time of the day, and additional stressors should be investigated.

## Data Availability

Data is available upon reasonable request.
